# Partitioning of multivariate phenotypes using regression trees reveals complex patterns of adaptation to climate across the range of black cottonwood (*Populus trichocarpa*)

**DOI:** 10.3389/fpls.2015.00181

**Published:** 2015-03-27

**Authors:** Regis W. Oubida, Dashzeveg Gantulga, Man Zhang, Lecong Zhou, Rajesh Bawa, Jason A. Holliday

**Affiliations:** Department of Forest Resources and Environmental Conservation, Virginia Polytechnic Institute and State UniversityBlacksburg, VA, USA

**Keywords:** *Populus trichocarpa*, black cottonwood, common garden, local adaptation, regression tree

## Abstract

Local adaptation to climate in temperate forest trees involves the integration of multiple physiological, morphological, and phenological traits. Latitudinal clines are frequently observed for these traits, but environmental constraints also track longitude and altitude. We combined extensive phenotyping of 12 candidate adaptive traits, multivariate regression trees, quantitative genetics, and a genome-wide panel of SNP markers to better understand the interplay among geography, climate, and adaptation to abiotic factors in *Populus trichocarpa*. Heritabilities were low to moderate (0.13–0.32) and population differentiation for many traits exceeded the 99th percentile of the genome-wide distribution of F_ST_, suggesting local adaptation. When climate variables were taken as predictors and the 12 traits as response variables in a multivariate regression tree analysis, evapotranspiration (Eref) explained the most variation, with subsequent splits related to mean temperature of the warmest month, frost-free period (FFP), and mean annual precipitation (MAP). These grouping matched relatively well the splits using geographic variables as predictors: the northernmost groups (short FFP and low Eref) had the lowest growth, and lowest cold injury index; the southern British Columbia group (low Eref and intermediate temperatures) had average growth and cold injury index; the group from the coast of California and Oregon (high Eref and FFP) had the highest growth performance and the highest cold injury index; and the southernmost, high-altitude group (with high Eref and low FFP) performed poorly, had high cold injury index, and lower water use efficiency. Taken together, these results suggest variation in both temperature and water availability across the range shape multivariate adaptive traits in poplar.

## Introduction

Tree species and populations may respond to climate change through migration, phenotypic plasticity, evolutionary adaptation, or local extinction (Aitken et al., 2008). Migration rates for trees—estimated to be less than 100 m per year (McLachlan et al., 2005)—are unlikely to be sufficient to keep pace with predicted rates of anthropogenic climate change (IPCC, 2007; Loarie et al., 2009), and adaptation to novel climatic conditions or phenotypic plasticity will, therefore, be necessary for persistence of local populations. Standing genetic variation in traits related to local adaptation increases the likelihood that such populations can make adjustments to mean phenotypes that enables persistence. Indeed, numerous studies have demonstrated that adaptive genetic responses to environmental change can occur in a relatively short period (a few years to hundreds of years), suggesting that local adaptation, at least in part, arises from standing variation rather than new mutations (Savolainen et al., 2013).

Understanding patterns of local adaptation across a species' range facilitates predictions of potential responses to climate change. Forest tree provenance trials that evaluate phenotypic differences among populations have identified traits under divergent selection and quantified their genetic bases (Sork et al., 2013). Such studies allow the (i) exploration of genotype by environment interactions (GxE), which is a necessary condition of local adaptation (Morgenstern, 1996); (ii) comparison of population differentiation in quantitative traits (Q_ST_) and neutral molecular markers (F_ST_), which can indicate the role of divergent selection in shaping adaptive genetic differences (Slavov and Zhelev, 2010); and (iii) detection of gradual phenotypic changes along geographical or environmental gradients (clinal variation), which is a signature of local adaptation.

*Populus trichocarpa* Torr. and Gray (black cottonwood) is a deciduous broadleaf tree species native to western North America that occupies riparian corridors with diverse topography and climate from California to southern Alaska (Gornall and Guy, 2007). Throughout most of its range, *P. trichocarpa* occurs close to the Pacific coast (within ~200 km), though it extends farther east in the Sierra Nevada Mountains of California and in northern British Columbia. The rapid growth of *P. trichocarpa*, ease of propagation, intraspecific variation, and substantial hybrid vigor for many important commercial and adaptive traits have contributed to its use for pulp, paper, and bioenergy (Zsuffa et al., 1996), and its potential use for carbon sequestration (Marron et al., 2005). Local adaptation in *Populus spp*. reflects the integration of many environmental factors—temperature, precipitation, photoperiod, wind, soil nutrient availability, growing season length, and biotic agents—which may be related to geographic variables such as latitude and altitude (Benowicz et al., 2000; Andersson and Fedorkov, 2004; Gornall and Guy, 2007; Hall et al., 2007; Vitasse et al., 2009; Keller et al., 2011; McKown et al., 2013). The strongest evidence of local adaptation in boreal and temperate forest trees is the synchronization of phenotypic traits with local photoperiod and seasonal temperature regimes (Aitken et al., 2008). However, evidence for local adaptation has been found for numerous other traits, including photosynthesis, branch characteristics, nitrogen-use efficiency, and water-use efficiency (Δ^13^C) (Gornall and Guy, 2007; McKown et al., 2013). Optimization of these traits in a particular environment may involve significant tradeoffs. Understanding these tradeoffs is a fundamental goal of ecological genetics, which may be advanced through multivariate analyses of phenotypes. While clustering and ordination provide homogeneous groupings with respect to multivariate phenotypes, they do not provide direct information on the environmental factors that drive these groupings. Multivariate regression trees (MRT) are an extension of classification and regression trees (CART) that enable such inference through the partitioning of response variables (e.g., phenotypic traits) according to multiple predictors (e.g., climate variables) (Hamann et al., 2011). In this study, we investigated genetic variation in 12 morphological, phenological, and physiological traits, and used MRTs to group these variables according to geography and climate to infer patterns of local adaptation across the range of *P. trichocarpa*. We also combine estimates of among-population trait differentiation (Q_ST_) with a null distribution of genome-wide neutral differentiation (F_ST_) to illustrate the potential role of divergent selection in shaping patterns of trait differentiation.

## Materials and methods

### Plant materials and common garden

**Figure 1:**
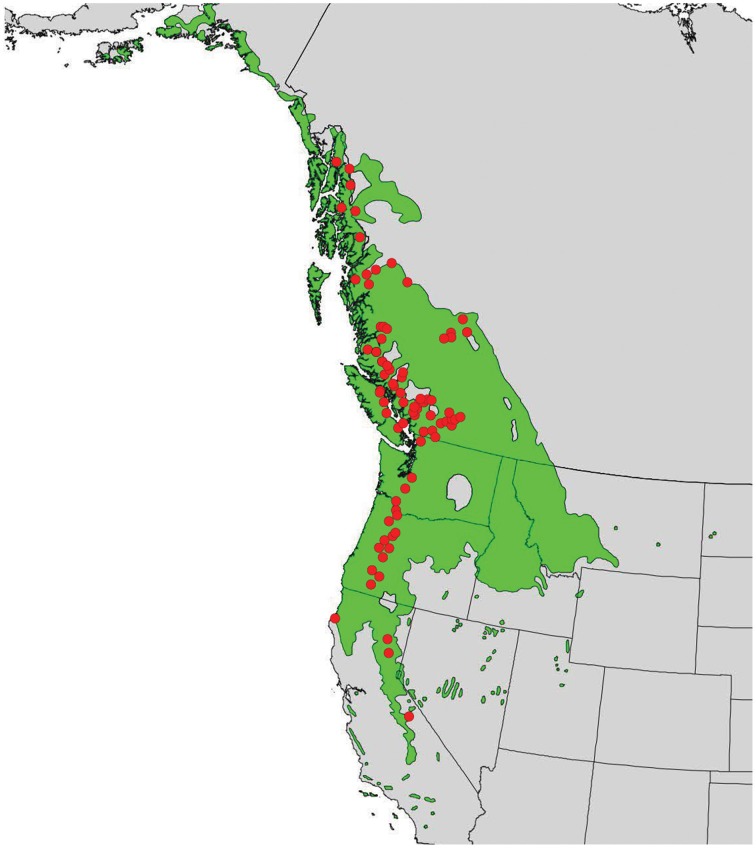
**Distribution of *Populus trichocarpa* (green shaded area) and origins of accessions described in this study (red points)**.

In 2010, branch cuttings of P. trichocarpa genotypes that span much of the species' range were collected and used to produce plantlets. The plantlets were grown for 6 months in a mist house and subsequently transplanted in May 2011 to an outdoor common garden located at the Reynolds Homestead Forest Resource Research Center located in Patrick County, Virginia (36°37′N and 80°09′W). Ramets of each genotype were planted in a randomized complete block design with 4 blocks. Annual climate variables (means of years 1981–2009) for the closest weather station (Patrick County, VA) showed a mean annual precipitation (MAP) of 1275 mm, a mean annual temperature (MAT) of 14.1°C, a mean warmest month temperature (MWMT) of 24.4°C, and a mean coldest month temperature (MCMT) of 4.2°C (obtained from the weatherbase.com). For this study, we selected 124 genotypes from the 789 available genotypes that covered a wide latitudinal and altitudinal gradient (37–58°N latitude and sea level through ~2300 m) (Figure 1).

### Phenotyping

#### Carbon isotope ratio

One branch-section from the current growth year was collected in March 2013 for each tree, oven-dried at 65°C until a constant weight was reached, and ground to 0.5 mm size. About 2 mg of each sample was combusted in an IsoPrime100 isotope ratio mass spectrometer (Isoprime Ltd, Cheadle Hulme, UK) located at the Virginia Tech Forest Soils and Biogeochemistry Laboratory. The carbon isotope ratio, ^13^C/^12^C (δ^13^C), was calculated relative to the international Pee Dee Belemnite standard (Farquhar et al., 1989) as follows:

$$\delta^{13}\text{C=}\frac{{\text{R}}_{\text{sa}}-{\text{R}}_{\text{sd}}}{{\text{R}}_{\text{sd}}}*1000[‰],$$

where R_sa_ and R_sd_ are the ^13^C:^12^C ratios of the sample and the standard, respectively (Craig, 1957).

#### Growth and branching

**Figure 2:**
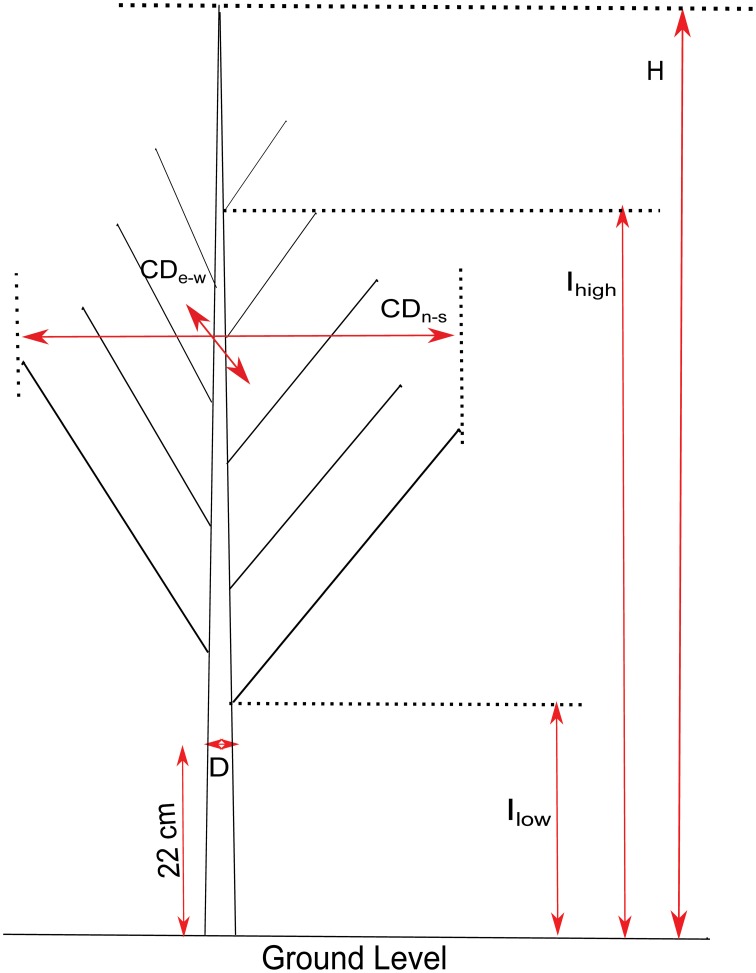
**Illustration of growth and branching traits**. H, Height; D, Stem diameter at 22 cm above ground level; CD, Crown Diameter (measured North-South and East-West); I_high_, insertion height of the highest branch; I_low_, height of the lowest branch.

In March 2013, while the trees were in a dormant state, growth and branch characteristics (Figure 2) were measured on the 124 genotypes. Height (H), crown diameter (CD), insertion height of the highest branch (proleptic or sylleptic) (I_h_), and insertion height of the lowest branch (I_l_) (Broeckx et al., 2012), were measured using a tape and a telescopic pole. Stem diameter (D) at 22 cm above ground level was measured using a digital caliper. Volume Index (VI = D^2^xH) was calculated according to Causton (1985). We also counted the total number of branches (NB) and the number of sylleptic branches produced during the 2012 growing season (NSyll) on the main stem of each tree. The relative number of branches (RNB = density of branches per unit of stem height) was calculated by dividing NB by H. CD was obtained by computing the mean of the crown diameters taken from two perpendicular directions (east to west and north to south). The relative canopy depth (RCD), defined as the percentage of the stem carrying branches, was computed as (I_h_–I_l_)/H.

#### Bud phenology and cold injury

Bud stage was scored on a weekly basis during April 2012 (BF = bud flush) and September 2012 (BS = bud set), and the date of BF or BS was converted to the Julian day (the number of days from December 31). The bud was considered flushed when a leaf had emerged 1 cm from the bud, and set when dark red/brown scales covered the apex. Buds that set or flushed before the first week or after the last week of scoring were recorded as having occurred 1 week before or 1 week later, respectively. Fall cold injury (I_−20_) was assessed by measuring electrolytic leakage of branch sections collected on November 5, 2012 (Hannerz et al., 1999). Cool (~4°C) temperatures were recorded at the site prior to sampling for cold hardiness, but no freeze event had yet occurred. For each genotype, three shoot segments (1–2 mm) were placed in solutions of 500 μl of distillated water with a trace amount of silver iodide to facilitate ice nucleation. Control samples were kept at 4°C. Test samples were placed in a Tenney programmable freezing chamber and the temperature was gradually lowered at a rate of 4°C/h until they reached –20°C, at which point they were incubated for 2 h and subsequently thawed at 4°C. An additional 500 μl of distilled water was then added and the samples were agitated on an orbital shaker to homogenize the solution. Conductivity was measured with a digital conductivity meter following the temperature treatments, and after heat-kill (95°C for 4 h). An index of freezing injury (I_t_) was calculated according to Flint et al. (1967):

$$I_{-20}=100(R_{t}-R_{0})/(1-R_{0});R_{t}=L_{t}/L_{k};R_{0}=L_{0}/L_{d}$$

Where *I_t_* is the index of injury (or percent injury), *R_t_* and *R*_0_ are the relative conductivities for –20°C and 4°C respectively, *L_t_* is the specific conductivity of leachate from the sample frozen at –20°C, *L_k_* is the specific conductivity of leachate from the sample frozen at –20°C and then heat-killed, *L*_0_ is the specific conductivity of leachate from control samples (4°C), and *L_d_* is the specific conductivity of leachate from the heat-killed control samples (Flint et al., 1967).

### Populations defined by multivariate regression trees (MRT)

Data were visually inspected for normality and homoscedasticity, and NSyll was linearized by log transformation before analysis. To reduce the number of variables, and to deal with the high correlation between growth and branching variables, principal component analysis (PCA) was run on the growth and branch traits using the *prcomp* function in R (R Core Team, 2014). MRTs were constructed using the R package MVpart v1.2–6 (http://cran.rproject.org/web/packages/mvpart/index.html). MRT is an extension of Classification and Regression Trees (CART) that can be used to explore, describe, and predict relationships between multivariate response data and multiple predictor variables (De'ath, 2002). A set of clusters are grown by repeated binary splits of the dataset to produce nodes as homogeneous as possible with respect to the response variables. The geographic and climatic variables were used as predictors and two principal components, BS, BF, I_–20_, and δ^13^C were used as response variables. For this analysis, 21 annual and seasonal climate variables (means of 1981—2009) were obtained using the ClimateWNA program (Wang et al., 2006) (v4.72). In addition, a drought index, the Standard Precipitation Evapotranspiration Index (SPEI), was obtained from the Global SPEI database (SPEIbase v.2.2). SPEI is calculated from the Climate Research Unit's TS3.2 database (http://www.cru.uea.ac.uk/data). Descriptions of each variable can be found in Supplemental Table 1.

### Estimation of genetic parameters

Samples were grouped into 20 populations according to their geographic origins (latitude, longitude, and altitude) for computation of population differentiation in quantitative traits (Q_ST_) and broad-sense heritability (H^2^). For each trait, the clonal best linear unbiased predictors (BLUPs) and variance components were estimated using restricted maximum likelihood (REML) with block as a fixed factor and genotype and population as random factors according to the following model:

$$Y_{ijk}=\mu +B_{i}+P_{j}+G_{k}(P_{j})+\varepsilon_{ijk},$$

where *Y*_*ijk*_ is the value for the *k*th genotype from the *j*th population in the *i*th block, μ is the population mean, *B*_*i*_ is the fixed effect of the *i*th block, *P*_*j*_ is the random effect of the *j*th population, *G*_*k*_(*P*_*j*_) is the random effect of the *k*th genotype in the *j*th population, and ε_*ijk*_ is the experimental error. Q_ST_ was computed as:

$$Q_{\text{ST}}=\frac{\sigma_{P}^{2}}{\left(\sigma_{P}^{2}+2\sigma_{G(P)}^{2}\right)}$$

Where σ^2^_*P*_ is the among population component of variance and σ^2^_*G*(*P*)_ is the genotype-within-population component of variance. These variance component estimates are based on clonal replication of trees, and have been shown by Lynch and Walsh (1998) to give Q_ST_ values confounded by non-additive genetic variance and maternal effects, which result in a slight deflation of Q_ST_. Hall et al. (2007) showed that Q_ST_ values estimated using this method probably represent lower bounds of Q_ST_. Therefore, our values are probably conservative with respect to adaptive population differentiation. Broad-sense heritabilities were calculated across-population (*H*^2^_*G*_) and within-population (*H*^2^_*G*(*P*)_):

$$H_{G(P)}^{2}=\frac{\sigma_{G(P)}^{2}}{\left(\sigma_{G(P)}^{2}+\sigma_{\varepsilon }^{2}\right)}$$

and,

$$H_{G}^{2}=\frac{\sigma_{P}^{2}+\sigma_{G(P)}^{2}}{\left(\sigma_{\left(P\right)}^{2}+\sigma_{G(P)}^{2}+\sigma_{\varepsilon }^{2}\right)}$$

Finally, genetic correlations between traits were obtained by calculating correlations among the respective BLUPs, and environmental correlations were estimated according to Searle (1961):

$$r^{\prime }=\frac{\text{R-r}\sqrt{H_{G}^{2}{}_{1}H_{G}^{2}{}_{2}}}{\sqrt{\left(1-H_{G}^{2}{}_{1}\right)\left(1-H_{G}^{2}{}_{2}\right)}}$$

Where R, r, and r′ are respectively the phenotypic, genetic, and environmental correlations, and *H*^2^_*G*__1_ and *H*^2^_*G*__2_ are the across-population heritabilities (*H*^2^_*G*_) of the relevant traits. Parametric bootstrapping was used to compute the 95% confidence intervals for the genetic parameters (Spitze, 1993; O'Hara and Merila, 2005) with 1000 bootstrap iterations of the variances components.

### Genotyping and population structure

We previously reported using sequence capture to retrieve and sequence much of the exome for 48 *P. trichocarpa* individuals originating from across the natural range (Zhou and Holliday, 2012; Zhou et al., 2014). These data have now been augmented with sequencing of an additional 391 samples. Capture, sequencing, assembly, and alignment are detailed in Zhou and Holliday (2012). Briefly, young leaf tissue was collected and genomic DNA was extracted using a Qiagen DNeasy Plant Mini Kit (Qiagen, Inc, Valencia, California, USA). A custom RNA bait library was designed and synthesized by Agilent Technologies (Santa Clara, California, USA), which targeted at least one exon for each gene model, 120 bp upstream of each gene model, as well as 1,000 random intergenic regions (at least 1000 bp from any annotated gene model). Our previous reports used a library comprised of 173,040 baits. Due to changes in the Agilent product, we were able to increase this to 230,720, a ~33% increase in the number of targeted regions. The data reported here includes only samples arising from this revised bait library. Captured libraries were sequenced on an Illumina HiSeq instrument. Following sequencing, the raw reads were trimmed of low quality bases, adapter sequences, demultiplexed using custom scripts, and aligned to the *P. trichocarpa* reference genome using BWA software (Li and Durbin, 2010). These reads were trimmed to retain the maximum length with an average quality score of 30 and a minimum of 13, with no undetermined nucleotides. For each genotype, SNPs were called for the uniquely mapped and re-paired reads using SAMtools software (Li et al., 2009) using a combined 8x cutoff of coverage depth and quality score of 30. A total of 1,058,513 SNPs were retained from the genomic regions that have 8x coverage in at least 75% of the 391 clones. Using the genotypes for which phenotyping is described in this paper, we estimated mean pairwise F_ST_ for the 727,993 segregating SNPs in that sample using the *hierfstat* R package according to the geographic groupings described above. To identify quantitative traits that may be under divergent selection, we compared Q_ST_ estimates with the empirical distribution of F_ST_, which was visualized using the *smooth.spline* function in R with a smoothing parameter of 0.6.

## Results

### Relationships among traits, geography, and climate

Correlations were computed for each pair of variables to evaluate the relationships among traits, geography, and climate. Among geographical variables, latitude had the highest number of significant correlations with climate variables (20/22 significant and 10/22 with |r| > 0.6) (Table 1). Longitude and altitude also had a moderate correlation with temperature and precipitation variables, but due to the southeast—northwest orientation of the species range, as well as the high altitude of the southernmost samples, it is difficult to disentangle latitude from longitude and altitude. The exception to this was the central part of the range (between 48° and 54°N Lat.), for which there was an increase in temperature differential (TD) and water deficit from the coast to the interior.

**Table 1:** **Pearson correlation coefficients and levels of significance between climate and geographic variables**.

**Climate variables**	**Geographic variables**		
	**Latitude**	**Longitude**	**Altitude**
Mean annual standard precipitation evapotranspiration index (SPEI12)	0.12	0.33^***^	−0.06
Mean annual temperature (MAT)	0.67^***^	−0.18	−0.43^***^
Mean warmest month temperature (MWMT)	0.74^***^	−0.43^***^	−0.14
Mean coldest month temperature (MCMT)	0.58^***^	0.01	−0.47^***^
Temperature difference between MWMT and MCMT (TD)	0.14	−0.39^***^	0.51^***^
Degree-days below 0°C (DD_0)	0.49^***^	0.03	0.50^***^
Degree-days above 5°C (DD_5)	0.71^***^	−0.25^**^	−0.35^**^
Degree-days below 18°C (DD_18)	0.65^***^	0.16	0.46^***^
Degree-days above 18°C (DD18)	0.72^***^	−0.31^***^	−0.13
Number of frost-free days (NFFD)	0.44^***^	0.03	−0.63^***^
Julian date on which FFP begins (bFFP)	0.51^***^	0.02	0.57^***^
Julian date on which FFP ends (eFFP)	0.48^***^	0.01	−0.57^***^
Frost free period (FFP)	0.50^***^	−0.01	−0.57^***^
Extreme minimum temperature over 30 years (EMT)	0.40^***^	0.03	−0.63^***^
Extreme minimum temperature (EXT)	0.76^***^	−0.53^***^	−0.04
Precipitation as snow (PAS)	0.41^***^	−0.03	0.47^***^
Mean annual precipitation (MAP)	0.30^**^	0.22^*^	−0.22^*^
Mean annual summer precipitation (MSP)	0.60^***^	0.42^***^	−0.14
Annual heat:moisture index (AHM)	0.43^***^	−0.22^*^	0.10
Summer heat:moisture index (SHM)	0.77^***^	−0.30^**^	0.08
Hargreaves reference evaporation (Eref)	0.89^***^	−0.50^***^	0.11
Hargreaves climatic moisture deficit (CMD)	0.80^***^	−0.40^***^	0.19^*^

Significant correlations are indicated as:

*** P < 0.001;

** P < 0.01;

* P < 0.05;

all other correlations are not significant.

All traits except RNB had a significant (*p* < 0.05) relationship with latitude of origin (Table 2). BS had the highest correlation with latitude (*r* = –0.79), while δ^13^C had correlations of 0.20 and –0.46 with latitude and altitude, respectively. The relationship with altitude was mostly driven by the southernmost group of samples, which had a low mean δ^13^C (–24.77) compared with the other populations. Among the traits, the highest correlations with climate were with temperature related variables (MAT, MWMT, MCMT, degree-days (DD), frost-free period (FF), EMT, EXT; Supplemental Table 2). BS showed the highest correlations with the climate variables, while δ^13^C had a weak negative but significant correlations with moisture variables (Hargreaves climatic moisture deficit (CMD): *r* = –0.32; Annual heat:moisture index (AHM): *r* = –0.22; Summer heat:moisture index (SHM): *r* = –0.27; Hargreaves reference evaporation (Eref): *r* = –0.28). Relatively strong positive genetic correlations (*r* > 0.50) were found between growth traits (H, D, VI) and branch traits (NB, NSyll and CD) (Table 3), with large trees (H, D, VI) generally displaying the highest NB and NSyll. I_–20_ was correlated with BS (*r* = 0.53) but there was no correlation between BF and either I_–20_ or BS. However, growth and branching traits had significant negative relationships with BF, and a positive relationship with BS and I_–20_. Finally, δ^13^C had had no significant correlations with the other traits.

**Table 2:** **Pearson correlation coefficients (r) and levels of significance calculated from genotypic means between traits and geographic variables**.

**Traits**	**Latitude**	**Longitude**	**Altitude**
H	−0.33^***^	−0.20^*^	−0.23^**^
D	−0.26^**^	−0.16	−0.25^**^
CD	−0.35^***^	−0.19^*^	−0.20^*^
NB	−0.32^***^	−0.21^*^	−0.18^*^
NSyll	−0.33^***^	−0.15	−0.13
RNB	−0.17	−0.14	0.03
RCD	−0.18^*^	−0.19^*^	−0.08
VI	−0.28^**^	−0.12^ns^	−0.24^**^
δ^13^C	0.20^*^	−0.04^ns^	−0.46^***^
BF	0.45^***^	0.47^***^	−0.09
BS	−0.79^***^	−0.39^***^	−0.03
I_−20_	−0.59^***^	−0.20^*^	−0.11

Significant correlations are indicated as:

*** P < 0.001;

** P < 0.01;

* P < 0.05;

all other correlations are not significant. Abbreviations: H, height; D, diameter; CD, crown diameter; NB, number of branches; NSyll, number of sylleptic branches; RNB, relative number of branches; RCD, relative canopy depth; VI, volume index; δ^*13*^C, carbon isotope ratio; BF, days to bud flush; BS, days to bud set; I_−*20*_, index of injury at −20°C.

**Table 3:** **Genetic (below diagonal) and environmental (above diagonal) correlations between traits with levels of significance**.

	**H**	**D**	**VI**	**CD**	**NB**	**Nsyll**	**RNB**	**RCD**	**δ13C**	**BF**	**BS**	**I–20**
H		0.90^***^	0.87^***^	0.88^***^	0.73^***^	0.59^***^	0.06	0.49^***^	0.25^**^	−0.48^***^	0.71^***^	0.83^***^
D	0.88^***^		0.96^***^	0.92^***^	0.81^***^	0.67^***^	0.20^*^	0.54^***^	0.23^**^	−0.43^***^	0.61^***^	0.74^***^
VI	0.82^***^	0.93^***^		0.89^***^	0.83^***^	0.71^***^	0.24^**^	0.54^***^	0.19^*^	−0.36^***^	0.61^***^	0.71^***^
CD	0.81^***^	0.89^***^	0.80^***^		0.82^***^	0.67^***^	0.29^**^	0.56^***^	0.18^*^	−0.49^***^	0.70^***^	0.81^***^
NB	0.65^***^	0.75^***^	0.69^***^	0.76^***^		0.87^***^	0.68^***^	0.75^***^	0.13	−0.45^***^	0.63^***^	0.63^***^
Nsyll	0.43^***^	0.56^***^	0.56^***^	0.53^***^	0.83^***^		0.57^***^	0.61^***^	0.01	−0.39^***^	0.62^***^	0.58^***^
RNB	0.21^*^	0.40^***^	0.31^***^	0.47^***^	0.81^***^	0.66^***^		0.67^***^	−0.12	−0.23^**^	0.21^*^	0.17
RCD	0.38^***^	0.50^***^	0.41^***^	0.51^***^	0.74^***^	0.59^***^	0.77^***^		0.04	−0.31^***^	0.37^***^	0.39^***^
δ 13C	0.05	−0.01	0.02	0.05	0.06	−0.04	0.02	−0.04		0.12	−0.14	−0.08
BF	−0.32^***^	−0.36^***^	−0.27^**^	−0.38^***^	−0.27^**^	−0.19^*^	−0.17	−0.12	0.17		−0.61^***^	−0.40^***^
BS	0.39^***^	0.34^***^	0.33^***^	0.32^***^	0.30^***^	0.33^***^	0.13	0.25^*^	0.01	−0.05		0.74^***^
I–20	0.26^**^	0.20^*^	0.17	0.26^**^	0.21^*^	0.13	0.1	0.24^**^	−0.01	−0.05	0.53^***^	

See Table 2 and methods for abbreviation definitions.

Significant correlations are indicated as:

*** P < 0.001;

** P < 0.01;

* P < 0.05;

all other correlations are not significant.

### Multivariate regression trees

**Figure 3:**
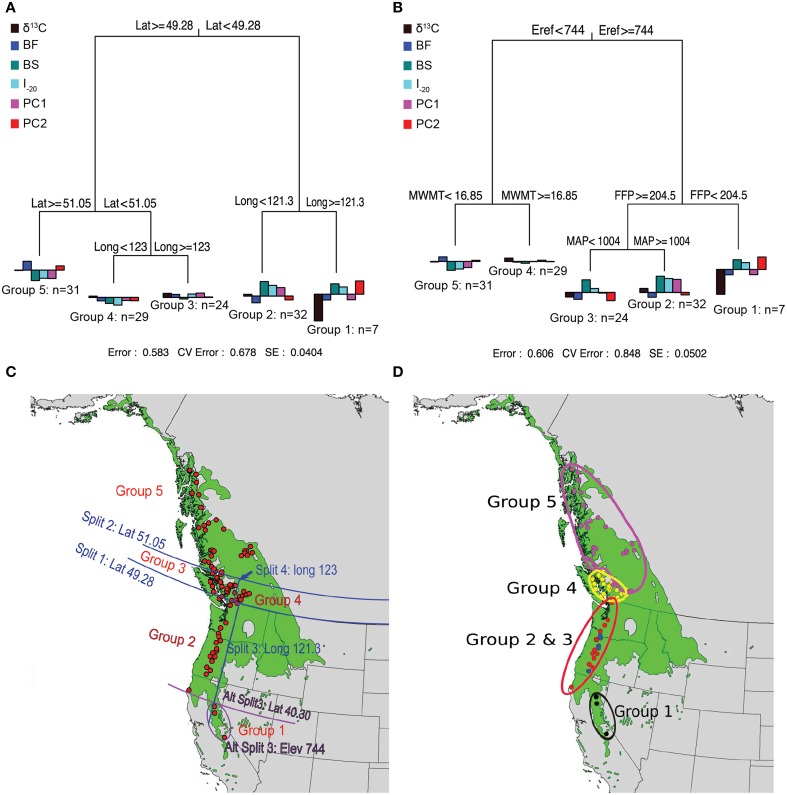
**Multivariate regression tree (MRT) analyses of the 124 black cottonwood genotypes with latitude, longitude and altitude as predictor variables (A); with climate as predictor variables (B); and geographic representation of the splits (C,D)**. Bar charts at the terminal leaves of the regression trees represent population means for the traits.

Prior to MRT analysis, growth and branching traits were subjected to PCA to remove their colinearity. The first two principal components were retained because they explained most of the variation in growth and branching traits (88%). The loadings of the traits (Supplemental Table 3) indicate that the first principal component (PC1) was positively correlated with all eight variables (0.56–0.95) whereas PC2 was mainly reflects branch characteristics (NB, NSyll, RNB, and RCD). The MRT using geography predictors split the 124 genotypes into five groups, which explained 42% of the variation in the traits (Figure 3). Trees originating from below 49.28°N Lat. were split into two groups according to their altitude of origin, a split that could also have been made based on latitude (40.30°N Lat.) or longitude (120.9°W Long.) with the same amount of variance explained. The seven southernmost genotypes (Group 1) had lower than average growth, fewer branches, and the lowest δ^13^C. This group also flushed early (small BF), set buds late (high BS), and had a high I_–20_. The 32 genotypes from Group 2, originating between 49.34° and 40.30°N Lat. (coastal), had above average growth and branching, small BF, high BS, and the highest I_–20_. Trees originating between latitudes 51.05° and 49.28°N Lat. (southern BC) were further differentiated into Groups 3 and 4 according to their longitude of origin. Group 4 (interior group) originated from areas east of 123°W and consisted of 29 genotypes characterized by growth (H, D, CD) slightly below the mean and low I_–20_, whereas the 24 genotypes from Group 3 (coastal group) originated from areas west of 123°W Long. and had growth and I_–20_ slightly above the sample mean. Group 5 consisted of 31 genotypes originating above 51.05°N. This group had the lowest growth, fewest branches, latest BF, earliest BS, and lowest I_–20_.

MRTs using climate variables as predictors also split the 124 genotypes in five groups, which explained 39% of variation in the measured traits (Figure 3). Group 1 contained seven high altitude genotypes, which originated from areas with Eref greater than 744 and FFP less than 204.5 days. These trees had low growth and branching, early BF, late BS and the lowest δ^13^C. Groups 2 and 3 originated from areas with Eref greater than 744 and FFP higher than 204.5 days, and were further split according to MAP, which revealed spatial heterogeneity in precipitation for this region. The 19 genotypes from Group 2 (MAP higher than 1004 mm) exhibited early BF, had the latest BS, the highest I_–20_, the highest performance in growth and branch characteristics, and the highest δ^13^C. The eight genotypes of Group 3 (MAP < 1004 mm) had growth and branch traits close to the average, the earliest BF, late BS, and high I_–20_. Group 4 had 29 genotypes originating from an area characterized by Eref < 744 and MWMT > 16.85°C, respectively. Genotypes from this population were characterized by growth and branching traits, δ^13^C, BS, BF, and I_–20_ close to the average. Finally, Group 5 was comprised of 31 genotypes that originate from localities with Eref lower than 744 and MWMT higher than 16.85°C. Genotypes from this group had the lowest growth and branch traits, early BS, late BF, and the lowest I_–20_.

### Population differentiation and heritability

**Figure 4:**
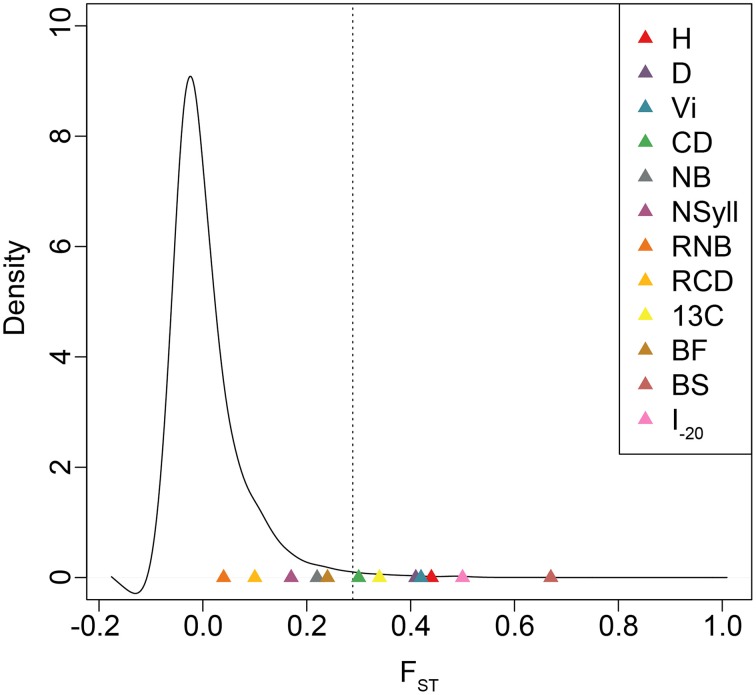
**Empirical distribution of F_ST_ based on 727,993 SNPs**. Dotted lines indicate the 99th percentile of F_ST_ values. Q_ST_ estimates for each trait are indicated with colored triangles.

BS and I_–20_ had the highest among-population differentiation, with Q_ST_ ≥ 0.5. Growth traits (H, D and VI) and δ^13^C had moderate values (0.32 ≤ Q_ST_ ≤ 0.44), while differentiation for branch traits (NB, NSyll, RNB and RCD) and BF was low (Q_ST_ < 0.25) (Table 4). BS, I_–20_, growth traits, and δ^13^C exceeded the 99th percentile of the empirical distribution of F_ST_, while the branching traits and BF fell below this threshold (Figure 4). Heritabilities (*H*^2^_*G*(*P*)_) were low to moderate (0.13 ≤ H^2^ ≤ 0.32; Table 4). BF, I_–20_, NB, and NSyll had the highest values (*H*^2^_*G*(*P*)_ ≥ 0.29), while VI had the lowest (*H*^2^ = 0.13).

**Table 4:** **Population differentiation in quantitative traits (Q_**ST**_) and broad sense heritability within-population (*H*^2^_*G*(*P*)_) and across the collection (*H*^2^_*G*_) for the 12 traits**.

	**Q_**ST**_**			***H***^**2**^_***G***(***P***)_			***H***^**2**^_***G***_		
**Trait**	**Value**	**Lower**	**Upper**	**Value**	**Lower**	**Upper**	**Value**	**Lower**	**Upper**
H	0.44	0.22	0.61	0.24	0.13	0.35	0.44	0.15	0.61
D	0.41	0.29	0.58	0.19	0.08	0.29	0.36	0.09	0.5
VI	0.43	0.25	0.59	0.13	0.02	0.24	0.26	0.01	0.39
CD	0.3	0.15	0.5	0.25	0.14	0.33	0.38	0.14	0.5
NB	0.22	0.09	0.34	0.32	0.2	0.43	0.43	0.23	0.52
Nsyll	0.18	0.05	0.31	0.29	0.19	0.43	0.37	0.17	0.48
RNB	0.04	−0.16	0.17	0.25	0.13	0.37	0.26	0.12	0.38
RCD	0.1	−0.08	0.24	0.21	0.1	0.33	0.25	0.11	0.36
δ^13^C	0.34	0.14	0.49	0.23	0.11	0.38	0.37	0.14	0.49
BF	0.24	0.1	0.4	0.3	0.18	0.4	0.41	0.17	0.57
BS	0.67	0.46	0.79	0.22	0.1	0.35	0.58	0.22	0.72
I_−20_	0.54	0.34	0.68	0.29	0.16	0.38	0.55	0.26	0.63

Estimates based on geographic populations described in methods. See Table 2 and methods for abbreviation definitions.

Lower/upper: lower and upper bounds of 95% confidence intervals.

## Discussion

### Local adaptation vs. drift

In spite of high gene flow among populations, temperate and boreal trees often display substantial adaptive genetic differentiation due to strong climatic gradients across their ranges (Farmer, 1996; Morgenstern, 1996; Howe et al., 2003; Savolainen et al., 2007; Aitken et al., 2008). In general, long-lived and widely distributed woody species harbor low levels of population differentiation in molecular markers, with mean F_ST_ often less than 0.10 (Hamrick et al., 1992). For *Populus*, in particular, F_ST_is even lower (<0.05; Slavov and Zhelev, 2010). Comparisons between F_ST_ and Q_ST_ are problematic when the distribution of the former, which may be influenced by demographic history, is not known (Whitlock, 2008). We, therefore, used a large, genome-wide sample of SNPs to estimate the empirical distribution of F_ST_ and determine the relative influence of drift and selection in generating observed patterns of trait differentiation across the range. Each of H, D, VI, CD, δ^13^C, BS, and I_–20_ fell outside the 99th percentile of the empirical distribution of F_ST_, which suggests that their differentiation is shaped by divergent natural selection.

*Populus* species exhibit strong latitudinal clines in bud set (Pauley and Perry, 1954), and the high values of Q_ST_ we found for bud set and cold injury are in agreement with estimates in range-wide studies of other woody taxa (Morgenstern, 1996; Hurme et al., 1997; Hall et al., 2007; Mimura and Aitken, 2007; Keller et al., 2011). Autumn phenology and cold acclimation are important targets of natural selection in northern tree species because they enable synchronization of annual growth cycle with the local climatic cycle to avoid cold damage while maximizing growth (Howe et al., 2003). Morgenstern (1969) and Keller et al. (2011) found high Q_ST_ values for BF (Q_ST_ = 0.52 in *Picea mariana* and 0.66 in *Populus balsamifera*), but most studies in woody species have reported low Q_ST_ for BF, in agreement with our results (Howe et al., 2003). This may reflect low genetic variation for the trait, but also the nature of many common garden studies, in which populations from across a latitudinal gradient are grown in an intermediate location. As bud flush is governed by both a chilling and heat sum requirement, it may be that when grown in such intermediate locations, trees from the north meet their heat-sum requirements earlier than they do at their origin (spring temperatures warmer than the population origin), whereas trees from the south meet that requirement later than at their origin (spring temperatures colder than the population origin). This hypothesis assumes that all populations receive adequate chilling. The high Q_ST_ value for bud set in *P. balsamifera* (Keller et al., 2011), the sister species to *P. trichocarpa*, may reflect the location of the common garden in that study, which was at the southern limit of the species range and included samples from across ~15° of latitude. Where among-population genetic differentiation in chilling or heat-sum requirements exists, such a common garden location may better reveal these differences. Further research using reciprocal transplants is needed to test this hypothesis. Finally, while height and diameter also exhibited strong differentiation consistent with local adaptation, Q_ST_ for branch traits fell below our empirical F_ST_ threshold, suggesting that any divergent selection related to branch traits is weak.

### Patterns of adaptation to local climate

Genotypes from the northern periphery of our sampling area had the lowest growth, which probably reflects adaptation to a short growing season (shorter FFP) in their native environment. For many tree species, common garden experiments have revealed that high latitude populations achieve less height growth even when they display higher assimilation rates than low latitude populations (Burtt, 1956; Kaurin et al., 1985; Junttila and Kaurin, 1990; Soolanayakanahally et al., 2009; Savage and Cavender-Bares, 2013). Interestingly, the southernmost genotypes (originating from between 1227 and 2363 m above sea level) had similar growth to those from the north, which could be explained by adaptation to the high altitude climate conditions in the Sierra Nevada Mountains of California. This area has low minimum temperatures and a short growing season (median EMT = –22.5 and FFP = 169.3) similar to the north (EMT = –25.8 and FFP = 179), and is much cooler than the common garden site. Climate variables related to water availability also reveal drier summers for the Sierra Nevada location (MSP = 159.2, Eref = 601, and CMD = 100) compared to the rest of the species range (mean rangewide MSP = 290.1, Eref = 728.8 and CMD = 273.3). Decreasing tree height with increasing altitude and latitude of origin has been described for a number of conifers and broadleaf trees (Rehfeldt, 1994; Oleksyn et al., 1998; Sáenz-Romero et al., 2006; Premoli et al., 2007; Rweyongeza et al., 2007; Vitasse et al., 2009). In addition to the tradeoff between growth and cold hardiness, possible explanations for the low growth of high altitude populations include high respiration rates, high allocation to roots, or reduced shoot-growth period (Oleksyn et al., 1998). At the same time, the high altitude genotypes from California had a relatively long growing season (early BF and late BS) in the common garden in spite of having a lower FFP (169 days) in their locality of origin. These two environments differ in temperature regime but not significantly in photoperiod (only 1° latitude), which suggests that the long growth period that occurred in the common garden was likely related to temperature differences. Indeed, the higher temperatures in the common garden compared to high altitude locations may have led to an earlier BF, which is mainly controlled by temperature (Luquez et al., 2008; Keller et al., 2011), although insufficient chilling may delay BF.

The center of the range (southern BC, Oregon, Washington) was partitioned into three populations. Genotypes from southern BC, which is characterized by moderate temperatures, high precipitation, and low evaporative demand, were differentiated into two groups according to continentality: the coastal group, which originates from a mild climate (MWMT > 16.85) with a long growing season, displayed better growth (slightly above the overall mean), and was more susceptible to fall cold damage than the interior group. The interior population in southern BC was merged with the northern groups in the MRT using climate predictors, as the temperature variables of these two population locations were similar. Genotypes spanning the coast of Oregon and Washington were the tallest, and had the highest number of branches in the common garden. This area is below 400 m altitude and has a mild climate (highest MAT, MWMT, EXT and low TD) that is similar to the common garden in terms of temperature variables. The climate variable-based MRT showed that these genotypes could be further differentiated according to MAP in spite of closely overlapping geography. This local spatial heterogeneity in climate could help these populations respond to climate change, facilitating migration by reducing dispersal distance required to find an appropriate climatic niche (Ackerly et al., 2010).

Plants adapted to arid environments generally have higher δ^13^C, an indirect proxy for water use efficiency (WUE), than plants adapted to wet environments (Passioura, 1982). By contrast, we found the lowest δ^13^C for genotypes originating from the most water-limited areas. Similar trends were observed in *Pseudotsuga menziesii, Larix occidentalis, Pinus ponderosa*, and *Pinus contorta* (Zhang et al., 1993; Zhang and Marshall, 1994, 1995; Aitken et al., 1995; Guy and Holowachuk, 2001), and may reflect differences in environmental conditions between common garden and population origins (Read and Farquhar, 1991; Aitken et al., 1995). Plants cope with fluctuation in water availability by adjusting their leaf area, root depth and density, hydraulic properties, photosynthetic capacity, and stomatal conductance. The adaptive strategy they use determines the strength and the sign of the correlation between δ^13^C and the climate (Aitken et al., 1995; Lauteri et al., 2004). The Sierra Nevada Mountains population (Group 1), which had the lowest δ^13^C, originates from a climate characterized by drier summers (MSP = 159 mm, AHM = 19.0, SHM = 135.8, Eref = 991) compared with the other populations (mean MSP = 328 mm, AHM = 13.3, SHM = 64.2, Eref = 681.5), for which δ^13^C values were higher. This may seem counterintuitive as one may expect genotypes adapted to dry locations would exhibit higher WUE (i.e., higher δ^13^C). However, when grown in the common garden, genotypes of Group 1 likely encountered a moister summer than in their native environment, and consequently may have adjusted one of the physiological parameters noted above, which may have resulted in lower δ^13^C. On the other hand, accessions from populations originating from areas with greater summer precipitation may have experienced mild water stress and responded by reducing their water loss, which led to higher δ^13^C. Aitken et al. (1995) found a similar result for douglas-fir (*Pseudotsuga menziesii*) seedlings grown at a location with intermediate precipitation compared with source populations (coastal and interior). This hypothesis is also supported by δ^13^C lower than the average for Group 3 in the climate-based MRT, for which summer moisture values (MSP = 138 mm, AHM = 21.4, SHM = 120.5, Eref = 865) were similar to Group 1.

### Relationships between productivity and adaptability traits

Branch characteristics are closely related to productivity as they determine the quantity of light interception and CO_2_ assimilation (Halle et al., 1978), which may explain the positive correlation between growth (H, D, VI) and branching variables. We found a higher number of sylleptic branches on highly productive genotypes, which is consistent with Ceulemans et al. (1990) and Scarascia-Mugnozza et al. (1999), who showed that sylleptic branches supply a larger proportion of carbon to the stem than proleptic branches in young poplar trees. The lack of a negative correlation between δ^13^C and growth/branch characteristics combined with moderate H^2^ for these traits suggests that, for *P. trichocarpa*, there is a possibility of improving WUE without compromising productivity. The continued development of poplars as bioenergy feedstocks depends partially on their suitability for cultivation on marginal lands that may be water limited (Berndes, 2002). Such a strategy would limit competition with food crops for the best sites. Hence, WUE will be a key target of selection of improved cultivars. Similar results were reported for interspecific poplar hybrids (Rae et al., 2004; Marron et al., 2005; Monclus et al., 2005, 2006; Fichot et al., 2010), *P. nigra* (Chamaillard et al., 2011), and *P. trichocarpa* (McKown et al., 2013). Farquhar et al. (1989) suggested that a lack of relationship between δ^13^C and growth means that stomatal conductance drives WUE variation, while a positive correlation between the two traits generally supports that differences in photosynthetic capacity is the main driver for WUE. Gornall and Guy (2007) showed that differences among *P. trichocarpa* genotypes in WUE were related to variability in stomatal conductance rather than assimilation rate.

## Conclusions

Our data suggest that *P. trichocarpa* has considerable standing adaptive variation within and among populations, which represent an adaptive potential. We found strong population differentiation in growth, phenology, cold hardiness, and carbon isotope composition, but weak genome-wide differentiation at SNP loci, which suggests that local adaptation better explains patterns of variation in these traits than drift alone. Multivariate regression trees using geography or climate variables as predictors showed that population differentiation in adaptive traits could be primarily explained by either latitude or evapotranspiration, respectively. The MRT analysis also suggested that the geographically intermingled accessions from coastal California and Oregon could be separated for the traits we measured on the basis of heterogeneity in mean annual precipitation. Genotypes from these areas were the best adapted to the climatic conditions of our Virginia common garden, suggesting that they can be used for breeding purposes in areas with similar environmental conditions. Indeed, with the growing interest in poplar species for bioenergy, knowledge of suitable genotypes for specific environments constitutes valuable information for successful plantations. One limitation of this study is the location of the common garden, which was located well outside the natural longitudinal range of *P. trichocarpa*, and at a latitude similar to the southern species range limit. In addition to the impacts of daylength regimes the common garden location likely had on growth and phenology, this environment may also impact the rankings of genotypes due to differences in soil conditions and temperature. In addition, fungal pathogens present in the southeastern US may impact performance of this species, particularly *Septoria musiva*, though we did not observe cankers during the course of this study. To address these issues, we have established an additional common garden site near the center of the species range, which will help to tease apart the relationship between genetics and environment.

### Conflict of interest statement

The authors declare that the research was conducted in the absence of any commercial or financial relationships that could be construed as a potential conflict of interest.
