# Supplementary material for: Partitioning of multivariate phenotypes using regression trees reveals complex patterns of adaptation to climate across the range of black cottonwood (Populus trichocarpa)
Source: Front Plant Sci. 2015 Mar 27;6:181. doi: 10.3389/fpls.2015.00181 (PMC4375981; doi:10.3389/fpls.2015.00181)
Supplement: Supplementary file 1 [file DataSheet1.DOCX]

Supplemental Table 1. Abbreviations of climate variables used in regression tree analyses.

**AH:M**: Annual heat:moisture index (MAT+10)/ (MAP/1000))

**bFFP**: The Julian date on which FFP begins

**CMD**: Hargreaves climatic moisture deficit

**DD<0 (DD_0)**: Degree-days below 0°C, chilling degree-days

**DD<18 (DD_18)**: Degree-days below 18°C, heating degree-days

**DD>18 (DD18)**: Degree-days above 18°C, cooling degree-days

**DD>5 (DD5)**: Degree-days above 5°C, growing degree-days

**eFFP**: The Julian date on which FFP ends

**EMT**: extreme minimum temperature over 30 years. For an individual year, the EMT is estimated for a 30-year normal period (one of the nine normal periods included in the package) where the individual year is nearest to the center of the normal period.

**Eref**: Hargreaves reference evaporation

**EXT**: extreme minimum temperature over 30 years. For an individual year, the EMT is estimated for a 30-year normal period where the individual year is nearest to the center of the normal period.

**FFP**: Frost Free Period (day)

**MAP**: Mean Annual Precipitation (mm)

**MAT**: Mean Annual temperature (°C)

**MCMT**: Mean Coldest Month Temperature (°C)

**MSP**: Mean Annual Summer P (May to Sept.) precipitation (mm),

**MWMT**: Mean Warmest Month Temperature (°C)

**NFFD**: The number of frost-free days

**PAS**: Precipitation as snow (mm). For an individual year, PAS is calculated for the period between August in previous year and July in current year.

**SH:M**: Summer heat:moisture index ((MWMT)/(MSP/1000))

**SPEI12**: Mean annual Standard Precipitation Evapotranspiration Index

**TD**: temperature difference between MWMT and MCMT, or continentality (°C)

Supplemental Table 2. Heat map with correlation coefficient between traits and climate variables

|  | | Morphology | | | | | | | | Physiology | Phenology | | |
| --- | --- | --- | --- | --- | --- | --- | --- | --- | --- | --- | --- | --- | --- |
|  |  | Growths | | | Branch pattern | | | | |  |  |  |  |
|  |  | H | D | Vi | CD | NB | NSyll12 | RNB | RCD | $\delta$^13^C | BF | BS | I_-20_ |
| Temperature | MAT | 0.48*** | 0.44*** | 0.46*** | 0.48*** | 0.45*** | 0.45*** | 0.16^ns^ | 0.25** | -0.02^ns^ | -0.33*** | 0,75*** | 0.62*** |
|  | MWMT | 0.42*** | 0.38*** | 0.39*** | 0.42*** | 0.40*** | 0.43*** | 0.17^ns^ | 0.23** | -0.17^ns^ | -0.41*** | 0,73*** | 0.48*** |
|  | MCMT | 0.41*** | 0.36*** | 0.40*** | 0.43*** | 0.41*** | 0.41*** | 0.16^ns^ | 0.21* | 0.04^ns^ | -0.22* | 0,67*** | 0.63*** |
|  | TD | -0.19 ^ns^ | -0.16 ^ns^ | -0.20* | -0.21* | -0.20* | -0.17 ^ns^ | -0.06^ns^ | -0.08^ns^ | -0.21* | -0.07 ^ns^ | -0,26** | -0.43*** |
|  | EMT | 0.47*** | 0.44*** | 0.45*** | 0.46*** | 0.43*** | 0.38*** | 0.12^ns^ | 0.23* | 0.21* | -0.19* | 0,54*** | 0.55*** |
|  | EXT | 0.39*** | 0.34*** | 0.35*** | 0.34*** | 0.33*** | 0.38*** | 0.09^ns^ | 0.17 ^ns^ | -0.15^ns^ | -0.39*** | 0,74*** | 0.47*** |
|  | DD_0 | -0.34*** | -0.32*** | -0.34*** | -0.35*** | -0.31*** | -0.31*** | -0.09^ns^ | -0.16^ns^ | -0.01^ns^ | 0.18* | -0,56*** | -0.54*** |
|  | DD5 | 0.50*** | 0.45*** | 0.48 | 0.50*** | 0.46*** | 0.48*** | 0.1 ^ns^ | 0.26** | -0.05^ns^ | -0.38*** | 0,79*** | 0.60*** |
|  | DD_18 | -0.48*** | -0.44*** | -0.46*** | -0.48*** | -0.44*** | -0.44*** | -0.16^ns^ | -0.25** | -0.01^ns^ | 0.31*** | -0,74*** | -0.63*** |
|  | DD18 | 0.40*** | 0.36*** | 0.39*** | 0.40*** | 0.38*** | 0.44*** | 0.15^ns^ | 0.21* | -0.18* | -0.38*** | 0,74*** | 0.48*** |
|  | NFFD | 0.48*** | 0.44*** | 0.46*** | 0.47*** | 0.43*** | 0.41*** | 0.13^ns^ | 0.24** | 0.15^s^ | -0.20* | 0,61*** | 0.58*** |
|  | bFFP | -0.52*** | -0.47*** | -0.50*** | -0.52*** | -0.46*** | -0.43*** | -0.14^ns^ | -0.25** | -0.15^ns^ | 0.27** | -0,68*** | -0.60*** |
|  | eFFP | 0.50*** | 0.46*** | 0.47*** | 0.51*** | 0.46*** | 0.41*** | 0.17^ns^ | 0.27** | 0.12^ns^ | -0.21* | 0,64*** | 0.61*** |
|  | FFP | 0.51*** | 0.47*** | 0.49*** | 0.52*** | 0.47*** | 0.43*** | 0.15^ns^ | 0.26** | 0.14^ns^ | -0.25** | 0,67*** | 0.61*** |
| Temperature + precipitation | AHM | 0.07 ^ns^ | 0.01 ^ns^ | 0.07 ^ns^ | 0.07 ^ns^ | 0.07 ^ns^ | 0.13 ^ns^ | 0.00^ns^ | -0.03^ns^ | -0.22* | -0.16 | 0.44*** | 0.21* |
|  | SHM | 0.25** | 0.17 | 0.20* | 0.24** | 0.21* | 0.27** | 0.06^ns^ | 0.06^ns^ | -0.27* | -0.33*** | 0.71*** | 0.50*** |
|  | Eref | 0.33*** | 0.27** | 0.30*** | 0.32*** | 0.31*** | 0.38*** | 0.14^ns^ | 0.16^ns^ | -0.28* | -0.40*** | 0,78*** | 0.54*** |
|  | CMD | 0.22* | 0.15 | 0.19* | 0.22* | 0.23*** | 0.30*** | 0.13^ns^ | 0.09 ^ns^ | -032** | -0.33*** | 0,72*** | 0.46*** |
|  | SPEI12 | -0.19* | -0.18^*^ | -0.16 ^ns^ | -0.12 ^ns^ | -0.04 ^ns^ | -0.02 ^ns^ | 0.09^ns^ | -0.05^ns^ | -0.17^ns^ | -0.13 ^ns^ | -0,17 ^ns^ | -0.06 ^ns^ |
| Precipitation | MAP | 0.00 ^ns^ | 0.06 ^ns^ | 0.01 ^ns^ | -0.01 ^ns^ | -0.04 ^ns^ | -0.06 ^ns^ | -0.05^ns^ | 0.02^ns^ | 0.16 ^ns^ | 0.13 ^ns^ | -0,31*** | -0.07 ^ns^ |
|  | MSP | -0.19* | -0.12 ^ns^ | -0.14 ^ns^ | -0.20* | -0.25** | -0.21* | -0.17^ns^ | -0.12^ns^ | 0.13^ns^ | 0.28** | -0,55*** | -0.31*** |
|  | PAS | -0.24* | -0.19* | -0.26** | -0.26** | -0.30*** | -0.32*** | -0.17^ns^ | -0.16^ns^ | 0.02^ns^ | 0.10 | -0.56*** | -0.50*** |

Strong correlation coefficient is indicated by dark red and weak correlation by Yellow. Significant correlations are indicated as: *** = P < 0.001; ** = P < 0.01; * = P < 0.05; n.s. = non-significant Traits are listed in order of category as follows: Morphology traits; Tree height, Stem diameter, Crown Diameter, Number of branch, Number of Sylleptic branches, Relative number of branch, Relative Canopy Depth. Ecophysiology traits; Carbon Isotope Ratio. Phenology traits; bud break, bud set, Cold Injury index (I_-20_).

Supplemental Table 3. Trait loadings of the first (PC1) and second (PC2) principal components of analysis on trees dimension and branch traits, with PC1 explaining 69.9% and PC2 explaining 17.8% of the cumulative variation in the data set.

| Trait | PC1 | PC2 |
| --- | --- | --- |
| H | 0.83 | -0.46 |
| D | 0.92 | -0.34 |
| Vi | 0.90 | -0.31 |
| CD | 0.91 | -0.25 |
| NB | 0.95 | 0.22 |
| NSyll | 0.82 | 0.27 |
| RCD | 0.74 | 0.46 |
| RNB | 0.56 | 0.78 |
